# Differences in Clinical Features and Diagnostic Strategies Between IgG4-Related Autoimmune Cholangitis and Cholangiocarcinoma

**DOI:** 10.3389/fonc.2021.540904

**Published:** 2021-03-18

**Authors:** Ke Zhu, Jin Yang, Ying-zhen Chen, Xue-rong Zhang, Xian-huan Yu, Jie Wang, Rui Zhang, Chao Liu

**Affiliations:** ^1^ Guangdong Provincial Key Laboratory of Malignant Tumor Epigenetics and Gene Regulation and Department of Biliary-Pancreatic Surgery, Sun Yat-sen Memorial Hospital, Sun Yat-sen University, Guangzhou, China; ^2^ Department of Hepatobiliary Surgery, The Affiliated Hospital, Southwest Medical University, Luzhou, China; ^3^ Department of Anesthesiology, Sun Yat-sen Memorial Hospital, Sun Yat-sen University, Guangzhou, China

**Keywords:** IgG-related autoimmune cholangitis, cholangiocarcinoma, diagnostic strategies, clinical features, surgical resection

## Abstract

IgG4-related autoimmune cholangitis (IgG4-AIC) is often difficult to distinguish from cholangiocarcinoma (CCA). This study aimed to determine a practical clinical strategy for distinguishing between IgG4-AIC and CCA to avoid unnecessary surgical resection. We retrospectively collected and compared the clinicopathological data between IgG4-AIC and CCA patients, including the clinical, serological, and radiological characteristics, to follow up on these patients to investigate the prognosis. Among the 377 patients who received surgical resection for suspecting CCA at the Sun Yat-Sen Memorial Hospital between June 2004 and June 2014, 14 patients were diagnosed as IgG4-AIC through histochemistry after surgery. Immunohistochemistry revealed that IgG4 was up-regulated in the plasma cells of IgG4-AIC tissues in 13 out of 14 patients. The serum CA19-9 level was significantly lower than in the CCA group. Patients with IgG4-AIC can only see slight or no enhancement under the contrast enhancement CT scan, while there are no signs of ring-like or delayed enhancement that is unique to CCA. Thirteen patients were followed up, and the time was 12 to 92 months. Three of them were regularly treated with prednisone after surgery, and original symptoms disappeared. Our study demonstrated that the combination of imaging with serum CA19-9 could improve the preoperative diagnostic value and reduce the rate of unnecessary resection.

## Introduction

IgG4-related autoimmune cholangitis (IgG4-AIC) is defined as an autoimmune disease involving the intro- and extrahepatic biliary tract, which belongs to the manifestation of IgG4-related disease (IgG4-RD) in the biliary tract (1, 2). About 25% of cases involve the gallbladder, so this is usually considered to be the same type of disease. Most IgG4-AIC patients manifest as elevated serum IgG4 levels (3, 4), and pathological examination shows chronic inflammation and concentric bile duct stenosis, with fibrosis and plasma cell infiltration as main changes (5). IgG4-AIC patients are sensitive to adrenal cortex hormone treatment (6). In 2012, new diagnostic criteria for IgG4-AIC were published, including histopathology, imaging, serology, concomitant diseases, and sensitivity to hormone therapy (Histology, Imaging, Serum IgG4, Other organ involvement, and Response to therapy), abbreviated to HISORt diagnostic criteria (7).

Cholangiocarcinoma (CCA) is a kind of epithelial malignant tumor derived from various locations within the biliary tract (8). CCA is categorized according to the anatomical locations as intrahepatic, perihilar, or distal cholangiocarcinoma. Due to the lack of symptoms at an early stage, most CCA patients have advanced-stage disease manifested as abdominal pain and jaundice (9, 10). Surgical resection is recommended as the mainstay of potentially curative therapy for all CCA subtypes (11, 12).

Due to plenty of overlapping characteristics between the two diseases, it’s hard to distinguish IgG4-AIC from CCA. IgG4-AIC is often misdiagnosed as a biliary malignant tumor, resulting in unnecessary surgical resection. To enhance the understanding of IgG4-AIC and avoid unnecessary surgery due to misdiagnosis, we have retrospectively collected cases of autoimmune cholangitis undergoing surgery in our hospital. In the present study, clinicopathological characteristics, serological examination, and radiological characteristics were used to describe and compare with CCA, and follow-up was performed to investigate efficacy and prognosis. Objective analyses of these data were performed, thereby increasing the accuracy of diagnosis and reducing unnecessary resection.

## Patients and Method

### Patients and Biliary Tract Tissue

Between June 2004 and June 2014, 377 patients who were suspected as CCA underwent surgical resection at the Department of Hepato-Pancreato-Biliary Surgery, Sun Yat-Sen Memorial Hospital, Sun Yat-Sen University (Guangzhou, China). Among these patients, 14 patients were verified as IgG4-AIC histopathologically after surgery, according to the HISORt diagnostic criteria (7). The paraffin-embedded biliary tract tissue specimen from each case was divided into two groups, including hematoxylin and eosin (HE) staining group and immunohistochemistry staining. Thirty-nine cases of patients with CCA were randomly chosen from the tissue bank of Sun Yat-Sen Memorial Hospital. No one received any chemotherapy or radiation therapy before surgical treatment. Written informed consent was obtained from every patient or their guardian.

### Immunohistochemistry

IgG4 immunohistochemical staining of bile duct tissue was completed according to the human immunoglobulin IgG4 immunohistochemical detection kit (Yaji, Shanghai, China) at the Medical Research Center of Sun Yat-Sen Memorial Hospital. The stained sections were independently evaluated by two investigators who were blind to the clinical data of patients. The immunohistochemical staining sections were first scanned under a light microscope at low magnification (x40), and then five non-overlapping fields were observed at a final magnification of x400 (13, 14). The sections were calculated by recording the number of IgG4-positive plasma cells in 10 high-power fields (HPFs, magnification, x400). The number of IgG4 positive plasma cells/HPFs was recorded and counted, and an average of 10 HPFs was taken. According to the 2012 Asian IgG4-related autoimmune cholangitis diagnostic criteria, when the number of IgG4-positive plasma cells/HPFs >10, it was considered diagnostically significant (7). When the suggestion of two observers was different, an agreement was reached by using a double-headed microscope.

### Abdominal Imaging

We have collected the first computer tomography (CT) or magnetic resonance (MR) of patients. CT or MRI scans were evaluated by a single radiologist, who was blind to the diagnosis, and recorded if there were any of the following abnormalities: biliary tract stenosis due to the thickened wall or tumor-like lesions in the bile duct, the dilation of the lumen, the enhancement of lesion, and peripheral lymphadenectasis.

### Follow-up

Only 13 patients have received complete follow-up. These 13 IgG4-AIC patients have received follow-up for 12–92 months (median, 30 months). For each patient, periodic reviews were recorded, including laboratory tests, abdominal ultrasound, CT, or MR scan. The deadline for the follow-up was December 2014.

### Statistical Analysis

Continuous variables were analyzed using Student’s t-test when the variables were normally distributed, like the age of patients, while the Mann-Whitney U-test was used when data did not follow the normal distribution, including ALT, AST, GGT, ALP, TBIL, CA19-9, CA125, AFP, and CEA. In all statistical analyses, P values were two-sided, and statistical significance was assumed with P<0.05. Statistical analyses were performed using SPSS 18.0 (SPSS Inc, Chicago, IL).

## Result

### IgG4 Expression in Bile Duct

**Figure 1:**
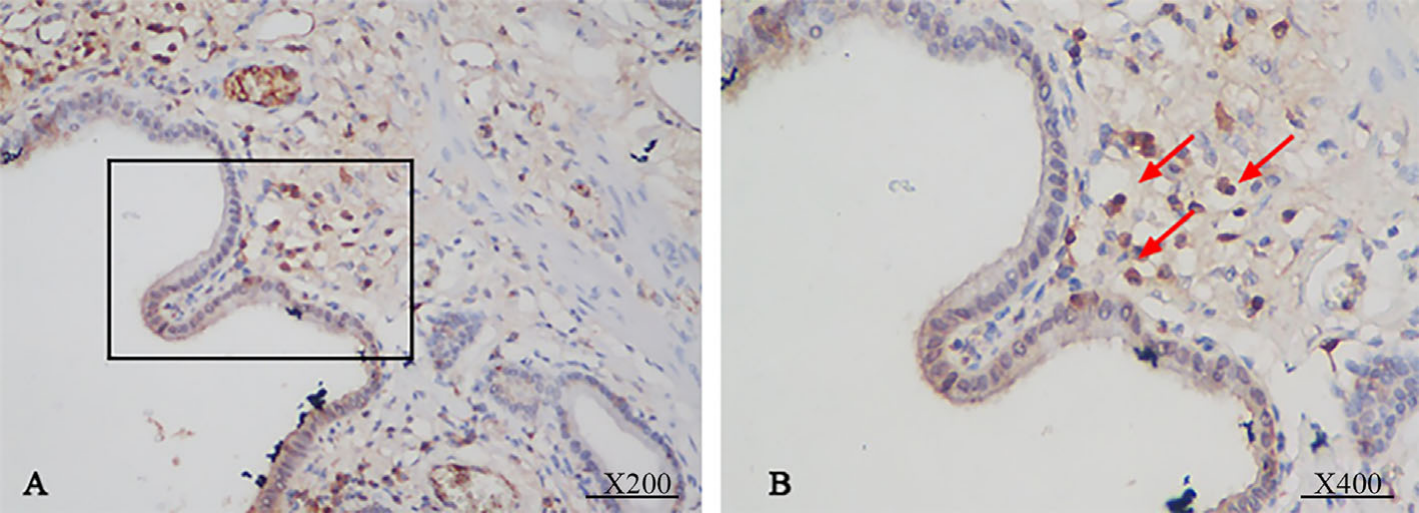
Immunohistochemical staining showed that the bile duct specimens were infiltrated by plenty of plasma cells. (**A**, magnification x200). The infiltrated plasma cells were stained positive for IgG4 more than 10 per 1 HPF (**B**, magnification x 400).

The immunohistochemistry staining of all bile duct tissues for IgG4 was performed to evaluate the expression of IgG4 (n=53). The stained sections showed that IgG4 was highly expressed in the plasma cells of IgG4-AIC tissues (92.9%, 13/14); the representative images are shown in **Figure 1**. On the contrary, IgG4 was not expressed in CCA tissues (0, 0/39).

### Pathology

**Figure 2:**
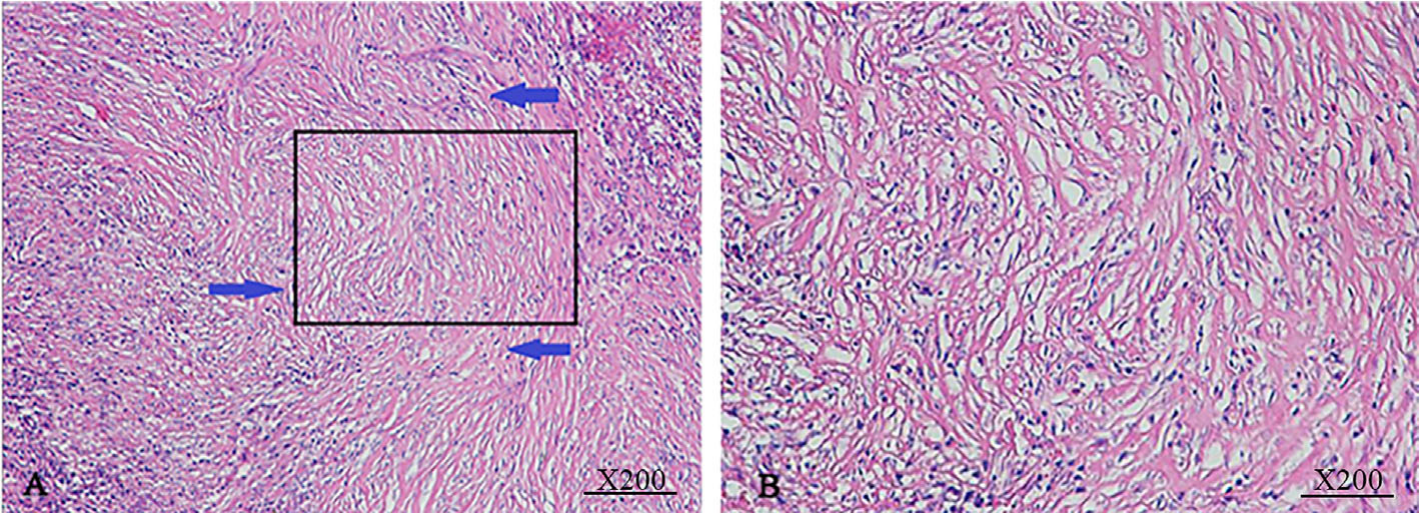
Macroscopic findings from IgG4-AIC specimens. IgG4-AIC tissues were routinely stained with H&E staining. The bile duct exhibited chronic cholangitis with fibrotic hyperplasia (**A**, magnification x100). These fibrous cells arranged disorderly and extended in all directions (**B**, magnification x200).

All IgG4-AIC patients with pathological confirmation (n=14) had specimens. HE staining showed microscopically detectable chronic cholangitis with storiform-arranged fibrosis: plasma cell infiltration (78.6%, 11/14), chronic lymphadenitis (71.4%, 10/14), seven had bile duct fibrosis (50.0%, 7/14); the representative images are shown in **Figure 2**. In addition, five patients were found to have lymph nodes swelling around the lesion during the operation, but these enlarged lymph nodes were confirmed to be inflammatory enlargement. No malignancy or dysplasia was verified in 14 cases of IgG4-AIC.

### Clinicopathological Data

The incidence of misdiagnosis of IgG4-AIC is 3.71% (14/377) in our center. The general characteristics of these patients were summarized in **Table 1**. Patients with IgG4-AIC manifested as abdominal pain (57.1%, 8/14), jaundice (50.0%, 7/14), emaciation (42.8%, 6/14), as shown in **Table 1**. Among seven patients with jaundice, four patients showed elevated serum CA19-9. In our study, there were five patients accompanied with autoimmune pancreatitis (35.7%, 5/14). The ratio of male to female is 9:5, and the mean of average onset age was 59 ± 12.8 years in IgG4-AIC patients (range, 41–79 years), which was significantly younger than in CCA (IgG4-AIC vs. CCA, 59 vs. 65; P=0.02). Among these 14 patients with IgG4-AIC, nine patients had an elevated level of ALT and AST, 12 presented an elevated level of TBIL and GGT, and 10 presented an elevated level of ALP. However, there were no statistically significant differences in ALT (P=0.770), AST (P=0.724), TBIL (P=0.211), GGT (P=0.593), and ALP (P=0.493) between the two groups. As for tumor markers, six patients showed increased serum CA19-9, two showed increased serum CA125, and only one showed increased serum AFP in patients with IgG4-AIC. This was significantly lower in patients with IgG4-AIC than in patients with CCA at the median serum levels of CA19-9 (26.3 vs. 228.5 kU L^-1^, P=0.025), which was observed as obviously elevated in CCA patients. However, there were no significant differences in CA125 (P=0.096) and AFP (P=0.572).

**Table 1:** Clinicopathological data of the IgG4-AIC patients.

Case No.	Coexistence of AIP^a)^	Biliary tract imaging	Histopathological		Laboratory examination						
			a/b/c/d^b)^	IgG4^+^/HPFs^c)^	Serum IgG4^d)^	AST U/L	ALT U/L	AFP μg/L	CEA μg/L	CA125 kU/L	CA19-9 kU/L
1	–	+	+/+/+/-	37	+	33.0	14.0	5.2	0.8	7.0	26.0
2	+	+	-/+/+/-	22	N/A^e)^	104.0	196.0	2.3	2.2	15.9	26.6
3	+	+	-/+/-/-	45	N/A	70.0	161.0	4.4	1.6	15.2	135.7
4	–	+	+/+/+/-	16	N/A	34.0	42.0	1.6	4.0	31.3	816.5
5	+	+	+/+/+/-	49	N/A	80.0	68.0	3.9	1.7	33.3	7.8
6	–	+	+/+/+/-	34	N/A	70.0	51.0	2.1	2.7	9.3	42.2
7	–	+	+/+/+/-	30	N/A	27.0	7.0	3.5	0.3	9.1	22.2
8	–	+	+/+/+/-	27	N/A	26.0	6.0	4.4	3.5	1.2	23.5
9	–	+	+/+/+/-	31	N/A	64.0	49.0	1105.0	1.8	87.1	52.6
10	–	+	+/+/+/-	29	N/A	45.0	29.0	1.5	5.5	15.2	55.9
11	–	+	+/+/+/-	36	N/A	100.0	130.0	3.9	7.8	31.6	2.1
12	+	+	+/-/+/-	6	N/A	63.0	54.0	2.9	2.9	37.6	4.4
13	+	+	-/+/+/-	48	N/A	165.0	268.0	4.4	4.1	10.7	416.9
14	–	+	+/+/-/-	41	+	20.0	13.0	3.0	2.3	10.4	12.5

^a)^AIP, autoimmune pancreatitis.

^b)^a, marked lymphocytic and plasmacyte infiltration and fibrosis; b, infiltration of IgG4-positive plasma cells: >IgG4-positive plasma cells/HPF; c, storiform fbrosis; d, obliterative phlebitis.

^c)^IgG4^+^/HPFs, the number of IgG4-positive plasma cells/HPFs in 14 IgG-AIC patients.

^d)^IgG4, immunoglobulin 4.

^e)^N/A, not applicable.

### Imaging Characteristic

**Figure 3:**
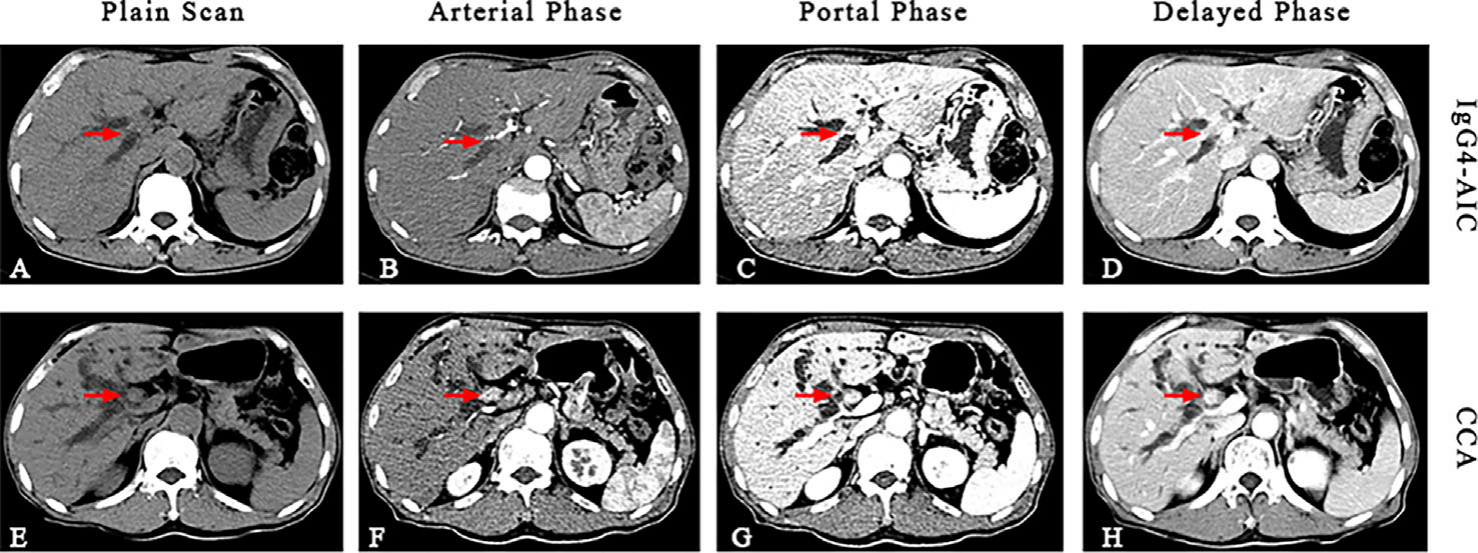
Comparison of IgG-AIC (upper) and cholangiocarcinoma (lower) during continuously enhanced scans. The image showed that IgG4-AIC has no obvious enhancement during the arterial phase, and then slightly irregular enhancement **(A–D)**. The CCA lesion has peripheral ring-like enhancement during the arterial phase, while the density of the central part is lower. During portal- and delayed-phase, the enhanced part showed centripetally expand as “delayed-enhancement” **(E–H)**.

**Figure 4:**
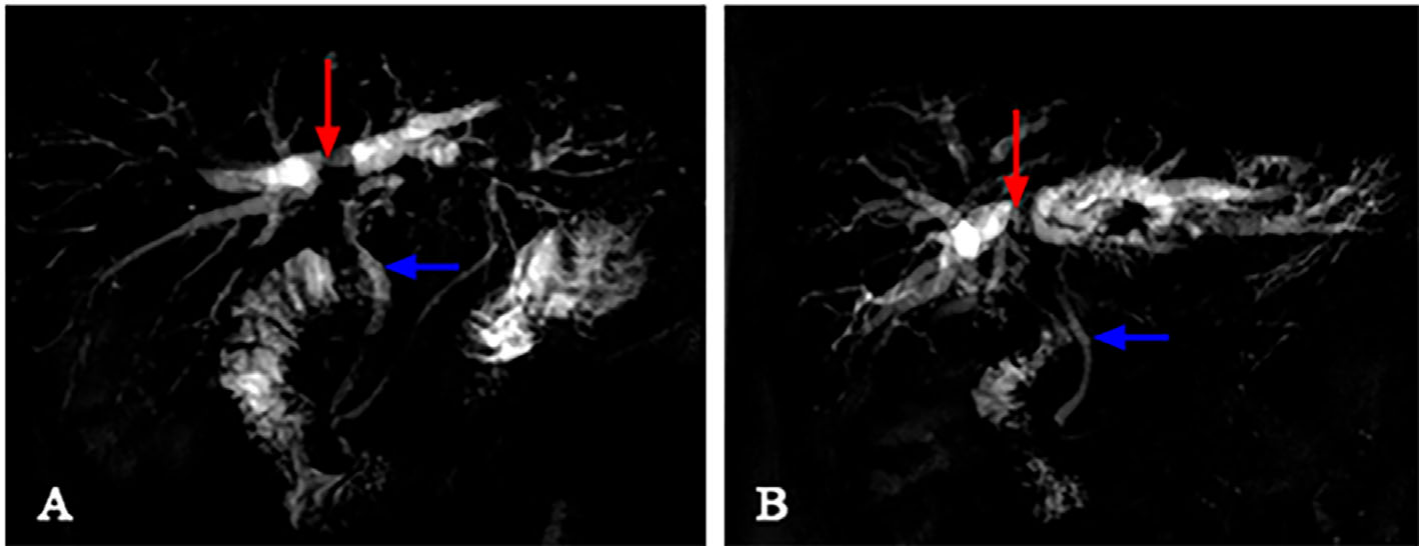
The differences between IgG4-AIC and CCA in MRCP. Above the obstruction site (red arrow), the intrahepatic bile duct dilatation of CCA revealed “soft rattan-like” change **(B)**, while IgG4-AIC was relatively stiff **(A)**. The normal bile duct adjacent to the lesion (blue arrow) presented smoother in CCA patients, while it presented as a “worm-like” change in IgG4 patients.

The image of IgG4-AIC patients showed that gallbladder wall or bile duct diffused thickened, stenosis of the bile duct, and dilation of the bile duct above the site of biliary obstruction. Out of these 14 patients, 5 exhibited enlarged lymph nodes that were misdiagnosed as lymph nodes metastasis (35.7%, 5/14). The most common involvement lesions found by imaging examination were the gallbladder in seven patients (50.0%, 7/14), followed by the common bile duct in five (35.7%, 5/14), the other intrahepatic bile duct in one (7.1%, 1/14), and the common hepatic duct in one (7.1%, 1/14). In addition, gallbladder wall thickening was found in seven patients, and four of them also presented thickened common bile duct. Through the enhanced scanning, the lesion was observed to exhibit low-density and no enhancement during the arterial phase in IgG4-AIC patients. By contrast, CCA tissues presented ring-like enhancement around and low-density in the center during the arterial phase, and enhanced area showed centripetal expansion as delayed-enhancement in the portal- and delayed-phase imaging (**Figure 3**). Magnetic resonance cholangiopancreatography (MRCP) revealed that common features were lumen stenosis of lesions and intrahepatic cholangiectasis above the lesion. In contrast to the sclerosis of the bile duct in IgG4-AIC patients, the dilatation of the intrahepatic bile duct is usually manifested as “soft rattan” or “crab foot” in CCA patients (**Figure 4**). The uneven thickness of bile ducts adjacent to the lesion area in IgG4-AIC patients showed an “insect-eroding” change. These differences in imaging were useful for differentiating IgG4-AIC from CCA.

### Prognosis and Response to Steroid

Thirteen out of 14 patients had received complete follow-up (92.9%, 13/14). The follow-up time was 12 to 92 months (median, 30 months). Ten patients presented intermittent abdominal pain after surgery (76.9%, 10/13), and seven of them were required to take a pain-killer (70.0%, 7/10). Three patients were treated with prednisone (0.6 mg/kg) after surgery, and their symptoms disappeared after treatment and no other organs have been found affected in these patients. Five patients were followed up in the outpatient clinic; new hepatic hilar lesions under CT imaging were found in one patient, intrahepatic diffuse disease through ultrasound examination was found in one patient, and an enlarged right kidney was found in one patient. The above three patients have discovered recurrence within 6 to 12 months after surgery.

## Discussion

IgG4-RD is a type of systemic disease that can involve any organ such as the pancreas, lungs, kidney, lymph nodes, and glands, which is characterized by infiltration of IgG4-positive plasma cells, elevated serum IgG4, and good response to the steroid, when it comes to the biliary tract is known as IgG4-AIC (15–17). On the resection specimens, IgG4-AIC shows characteristic histology and positive IgG4 immunostaining, present that infiltrated IgG4-positive plasma cells with storiform fibrosis and/or obliterative phlebitis in the thickened biliary tract wall (18).

CCA is the second most common primary liver cancer that originates from the epithelial lining in any location of the biliary tract. Even though patients with CCA have received surgical resection, 5-year survival is still very low (19). In the current study, IgG4-AIC patients were misdiagnosed as CCA resulting in major surgical resection, and it is challenging to distinguish IgG4-AIC from CCA, especially on the clinical symptom or serological examination (20). Serum IgG4 concentration is regarded as a useful examination in diagnosis (5, 21, 22). However, serum IgG4 has not been a routine examination in most hospitals as yet. Increased serum IgG4 cannot be the golden standard of IgG4-AIC, as it could also appear in extrahepatic CCA or some other non-IgG4-RD (23, 24). Unlike serum IgG4, biochemical indicators such as ASP, ALP, GGT, ALP, and tumor markers, such as CA19-9, CA125, and CEA, were routinely examined before surgery. In the current study, 14 patients were misdiagnosed as malignant tumors, and all underwent surgical resection. We found that the average onset age of IgG4-AIC is 6 years younger than CCA. Notably, only 35.7% of patients presented autoimmune pancreatitis, which is far lower than in similar studies abroad (9, 25–27). The serological examination showed that the serum level of ALT, AST, TBIL, GGT, and ALP was elevated, which has no statistically significant difference between IgG4-AIC and CCA. In our study, the level of serum CA19-9 in CCA was significantly higher than in IgG4-AIC. Thus, serum CA19-9 level could be a useful biomarker, because a level higher than 100kU L^-1^ is less in IgG4-AIC than in CCA [IgG4-AIC vs. CCA, 21.4% vs. 60%–80% (28–31)]. Some researchers suggested that CCA should be suspected when the level of serum CA19-9 was above 129kU L^-1^. Therefore, we set the cutoff value of CA19-9 below 129kU L^-1^, the sensitivity of imaging combined with serum CA19-9 to diagnose IgG4-AIC is 78.6%, and the specificity is 84.6%. Unfortunately, the level of serum IgG4 was not performed in our study, so we could not evaluate the value of serum IgG4 in diagnosis.

As for the abdominal, it has been reported that 96% of patients with IgG4-AIC showed characteristic image findings, manifested as multifocal strictures, thickened biliary duct, and peripheral lymphadenectasis (32, 33); these features of IgG4-AIC result in confusing it with CCA. In addition, imaging abnormalities of CCA include solitary lesion, eccentric biliary tract thickening, and obstruction of the involved segment (34). In our study, the involved gallbladder or bile duct showed that the tract wall was thickened, the lumen was stenosis, and the biliary dilatation upstream of the lesion in IgG4-AIC patients. Images of patients with IgG4-AIC have no or mild enhancement during contrast-enhanced CT scan different from ring-like or delayed enhancement in CCA.

In past research, steroid therapy was considered as routine treatment for nearly all IgG4-RD patients, and symptoms could be typical indications such as abdominal pain or jaundice (6, 21). The current standard treatment after surgery is 0.6 mg/kg of prednisone for 2–4 weeks, and then gradually reduced to 5 mg/day within 3 to 6 months. After that, long-term maintenance treatment with a low dose of prednisone (2.5–5 mg/day) for 3 years is recommended to prevent relapses (35). However, there was no prospective data or randomized controlled trial to judge whether it was safe to take long-term maintenance. Severe steroid-related complications were found in patients with IgG4-RD after 3 years of steroid treatment, such as decompensated cirrhosis or osteoporotic fractures (32, 36). Another steroid therapy protocol is to treat with 40 mg/day of prednisone for 4 weeks and then 5 mg/week for 11 weeks (31). In our study, the response to steroid therapy was excellent. Maintenance steroid therapy was performed in 3 patients, and they were found to have symptoms relieved. The follow-up found that even if the IgG4-AIC lesion has been resected, IgG4-RD may still recur in other organs owing to the characteristic of systemic disease. Thus, the treatment protocol with steroids was necessary for patients with IgG4-AIC.

Our study summarized the clinical, serological, radiological characteristics of 14 IgG4-AIC patients, and it provides the basis for the diagnosis and differential diagnosis. Besides, optimizing the clinical diagnosis to distinguish IgG4-AIC from CCA to reduce misdiagnosis and decrease unnecessary surgery was recommended based on our study. For patients who cannot distinguish between IgG4-AIC and CCA, the surgeon could conduct multidisciplinary treatment with radiologists and oncologists, and consider whether the patient needs surgical resections from the perspectives of clinical symptoms, serology, and imaging. For patients suspected of having CCA, patients with obviously elevated serum CA19-9 are more likely to suffer malignant tumor that needs to receive surgical treatment to perform the biopsy. More attention should be paid to imaging data if the serum CA19-9 level increases slightly, and serum IgG4 should be performed. Patients with slightly increased CA19-9 and increased IgG4 are regarded as IgG4-AIC patients, while those patients without increased serum IgG4 are suggested to receive diagnostic steroid treatment to avoid unnecessary surgical resection. Steroid therapy is recommended to be routine treatment, which is conducive to eliminating symptoms but also could effectively prevent a recurrence.

## Conclusion

In summary, IgG4-AIC is a special type of cholangitis, belonging to IgG4-RD. As the clinical manifestations of IgG4-AIC are similar to biliary tract malignant tumors, it’s hard to distinguish IgG4-AIC from those tumors, which leads to unnecessary surgical resection. In our study, we retrospectively compared clinicopathological characteristics, the follow-up of IgG4-AIC and CCA patients in our hospital, and demonstrated that combination of imaging with the level of serum IgG4 and CA19-9 could improve preoperative diagnostic sensitivity and specificity, and reduce misdiagnosis to reduce unnecessary surgical treatment.

## Data Availability Statement

All datasets generated for this study are included in the article/supplementary files.

## Ethics Statement

The studies involving human participants were reviewed and approved by the ethics committee of Sun Yat-sen Memorial Hospital, Sun Yat-sen University. The patients/participants provided their written informed consent to participate in this study.

## Author Contributions

KZ, JY, RZ, and CL contributed to the conception and design of the study. KZ and JY performed the experiments. Y-ZC and X-RZ collected and organized the data. X-HY and JW performed the statistical analysis. KZ and JY wrote and edited the manuscript and figures. RZ and CL reviewed the manuscript and provided feedback. KZ revised and edited the manuscript. All authors contributed to manuscript revision, and read and approved the submitted version.

## Funding

This work was supported by The Special Research Foundation of the National Nature Science Foundation of China (81972262,81972255); the Guangdong Natural Science Foundation (2020A1515010117, 2018A030313645, 2016A030313840, 2015A030313101); the Fundamental Research Funds for the Central Universities (18ykpy22); Grant [2013]163 from Key Laboratory of Malignant Tumor Molecular Mechanism and Translational Medicine of Guangzhou Bureau of Science and Information Technology; Grant KLB09001 from the Key Laboratory of Malignant Tumor Gene Regulation and Target Therapy of Guangdong Higher Education Institutes; and Grant from Guangdong Science and Technology Department (2015B050501004, 2017B030314026).

## Conflict of Interest

The authors declare that the research was conducted in the absence of any commercial or financial relationships that could be construed as a potential conflict of interest.
